# Multi-regional sequential pain syndrome

**DOI:** 10.3389/fneur.2025.1553455

**Published:** 2025-04-28

**Authors:** Xiao Yang, Kai-Jun Zhao, Jian-Min Liu

**Affiliations:** ^1^Department of Neurosurgery, Shanghai East Hospital, School of Medicine, Tongji University, Shanghai, China; ^2^Neurovascular Centre, Changhai Hospital, Naval Medical University, Shanghai, China

**Keywords:** multi-regional sequential pain syndrome, hypoperfusion, cryptogenic vertebral artery dissection, pain, vertigo

## Abstract

**Objective:**

To report a novel clinical entity, “Multi-regional Sequential Pain Syndrome” (MRSPS), and evaluate the relationship between hypoperfusion, cryptogenic vertebral artery dissection (CVAD), and MRSPS.

**Methods:**

A 59-year-old female patient, with a chronic MRSPS condition spanning 10–30 years, underwent a comprehensive diagnostic evaluation including cranial CTA, MRI, DSA, and CT perfusion, culminating in the identification of CVAD via dynamic contrast-enhanced computed tomography (DCE-CT). The treatment strategy integrated repairing CVADs with stent implantation to address the hypoperfusion and MRSPS.

**Results:**

Following intervention, hypoperfusion achieved complete improvement, and the patient achieved the complete resolution of pain and vertigo, with the modified Rankin Scale (mRS) score of 0 at the 1-year follow-up, signifying full neurological recovery.

**Conclusion:**

CVADs, a key cause of brain tissue hypoperfusion in MRSPS, can be effectively treated by repair to alleviate the syndrome.

## Introduction

Pain significantly impairs patients’ sleep (1–3), psychology (4), cognition (5), immune responses in the nervous system (6), and overall quality of life, including work and daily activities. Therefore, effective and timely pain control is essential. However, the pathophysiology of pain is complex and often unclear, resulting in palliative treatments being the primary option in most cases (4). It is noteworthy that a type of widespread, serial pain involving multiple areas in a top-down manner has not yet been named or reported. In this study, its etiologies, mechanisms, diagnosis, and treatment are being thoroughly investigated and discussed for the first time.

## Materials and methods

### Case presentation

The patient, a middle-aged woman, has been enduring headaches, scalp pain, and facial pain for the past 36 years. Notably, these symptoms have been significantly aggravated by physical contact, particularly when her scalp and face are touched or during the process of washing. She has been taking over-the-counter pain medications for treatment. Twenty years ago, the patient developed additional pain in the neck, right shoulder, both rib cages, chest area, both sides of the abdomen, waist, and stomach. This pain, described as a series of linked sensations, could be triggered by touch, turning over, and consuming cold meals, milk, rice, and mixed congee, with poor relief from various pain medications and significant sleep disruption. In the last decade, the patient has also experienced persistent dizziness and vertigo that alternate, with head turning capable of inducing dizziness, sometimes leading to sudden falls. At night, during sleep, she often feels a spinning sensation or as if falling from a height. In the past 3 months, the series of linked pains have worsened, transitioning from intermittent to continuous, with pain medications becoming less effective. The patient has been treated for conditions such as neuralgia, disk herniation, and gastritis outside, with suboptimal outcomes.

### Diagnosis

1. Chronic pain
2. Vertigo
3. Stomachache
4. Cryptogenic vertebral artery dissection
5. Multi-regional sequential pain syndrome

### Test method

In this study, due to the patient’s presence of pain in multiple areas, the onset of pain occurred in a top-down sequence, with progressive exacerbation of the pain, and was accompanied by other intractable symptoms, such as stomachache, alternating episodes of dizziness and vertigo, and so on. Therefore, we firstly termed this clinical presentation as the “Multi-regional Sequential Pain Syndrome (MRSPS), as well confirmed in the following results.

**Figure 1:**
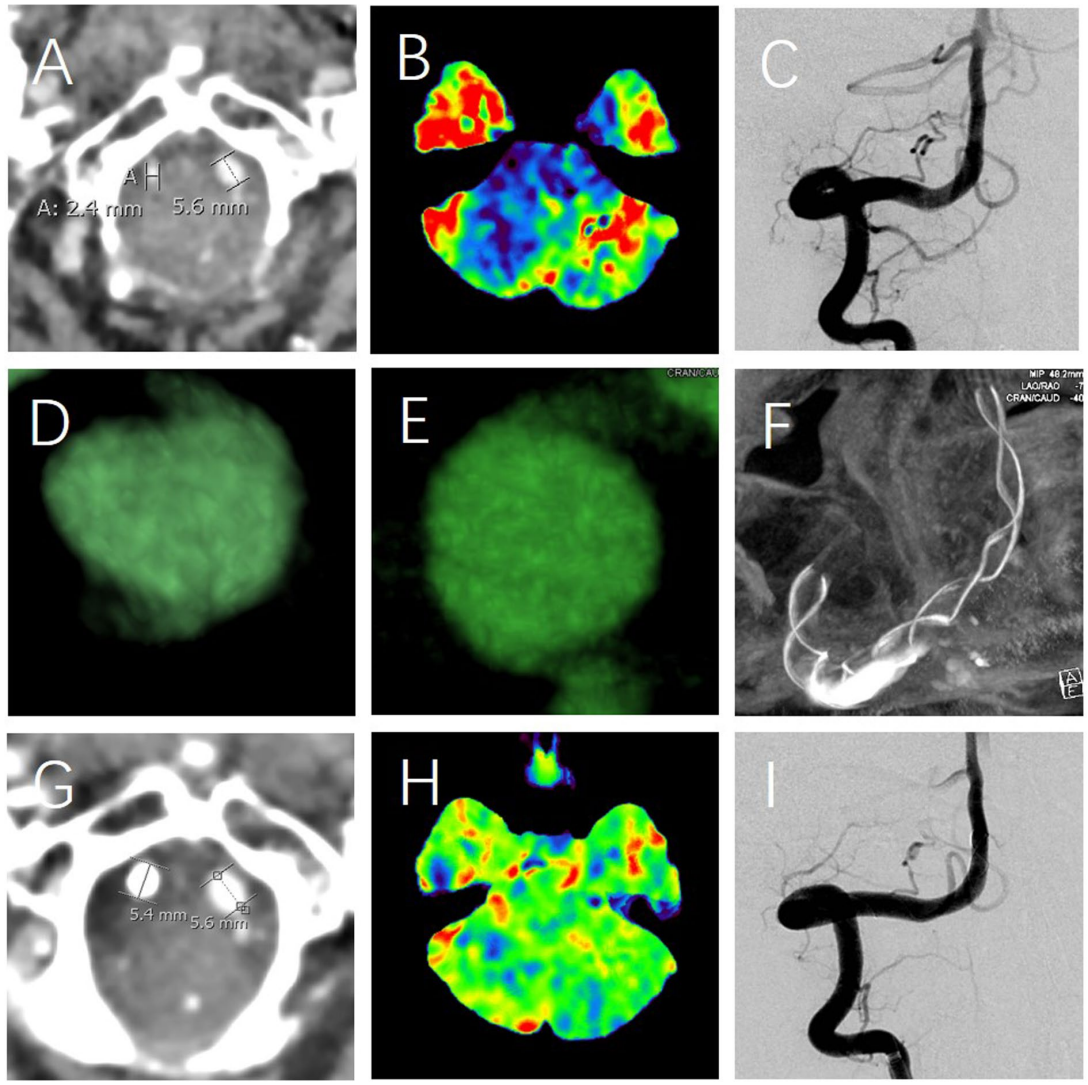
The diagnosis and treatment of multi-regional sequential pain syndrome. The first abnormality observed is a smaller right vertebral artery **(A)**, coupled with a relatively better perfusion of posterior circulation (blue, **B**). DCE-CT identified the right CVAD, characterized by the normal vascular morphology **(C)** and abnormal intimal flaps (white arrow, **D,E**), which was subsequently addressed using a stent **(F)**; The second significant abnormality observed was as follows: despite DSA showing no change in the morphological appearance of the vertebral artery (**C** vs. **I**), there were considerable changes detected in the vascular status (**A** vs. **G**) and perfusion (**B** vs. **H**) levels before and after the treatment. Specifically, the vascular status **(A)** and perfusion **(B)** exhibited marked differences when juxtaposed with their post-treatment counterparts in **G** and **H**, respectively.

Despite the absence of significant abnormalities in routine CT angiography (CTA, Figure 1A), magnetic resonance imaging (MRI), and digital subtraction angiography (DSA), cranial CT perfusion revealed hypoperfusion in the posterior circulation and bilateral temporal lobes (Figure 1B). Further examination with dynamic contrast enhanced computed tomography (DCE-CT) revealed CVAD in both vertebral arteries (Figures 1C–E) (7), with the right side being more severely affected (Figures 1C,D). The patient’s right vertebral artery exists a relatively smaller vascular diameter (Figure 1A), with relatively poor perfusion in the posterior circulation and the right temporal lobe (Figure 1B). Meanwhile, the right CVAD remains relatively longer. Especially, the most important characteristic of these dissections is that they are concealed within arteries that appear normal in morphology (7).

### Treatment and follow-up

The treatment strategy is to prioritize the elimination of the right CVAD. Therefore, on the basis of adequate dual antiplatelet medications, a stent reconstruction of the CVAD of the right vertebral artery was successfully performed under general anesthesia. Attentively, this form of dissection has also been effectively addressed in previous treatments (7). Throughout the perioperative period and during the postoperative 1-year clinical follow-up, the patient’s MRSPS has completely resolved (Table 1), allowing for the consumption of foods that previously triggered stomach pain without any issue. The patient’s symptoms of dizziness and vertigo have completely resolved, and the modified Rankin Scale (mRS) score was 0 at 12-month follow-up.

**Table 1:** Patient symptoms, onset time, and changes before and after intervention.

Symptoms	Onset time	Before intervention	After intervention				
			Worsened	No change	Partial improvement	Completely resolved	TPOSR
Headaches*	30 Y	NR				+	IAO
Scalp pain*	30 Y	GW				+	IAO
Facial pain*	30 Y	GW					IAO
*Triggered by physical contact (i.e touch)	30 Y	GW				+	IAO
Neck pain*	20 Y	GW					IAO
Right shoulder*	20 Y	GW				+	IAO
Both rib cages*	20Y	GW				+	IAO
Chest area*	20 Y	GW					IAO
Both sides of abdomen*	20 Y	GW				+	IAO
Waist pain*	20 Y	NR				+	IAO
Stomach pain^#^	20 Y	GW				+	IAO
*#Triggered by physical contact* and consuming cold meals#	20-30Y	GW				+	IAO
Syncope^§^	10 Y	NR				+	IAO
Vertigo^§^	10 Y	GW				+	IAO
Dizziness^§^	10 Y	NR				+	IAO
^§^Triggered by head turning	10 Y	NR				+	IAO

Years, Y; No relief, NR; Gradually worsening, GW; Time to postoperative symptom relief (TPOSR): Immediately after operation (IAO), before discharge (BD), after discharge (AD). * indicates somatic pain; # indicates visceral pain; § indicates symptoms of dizziness/vertigo/ syncope.

## Discussion

The case presented in this study is a striking illustration of a previously uncharacterized clinical phenomenon, which we have termed MRSPS. The patient’s long-standing and progressively worsening pain, coupled with the alternating episodes of dizziness and vertigo, presented a complex diagnostic and therapeutic challenge. This discussion aims to explore the possible etiology, the rationale behind the treatment strategy, and the implications of outcomes.

### Etiology

The etiology of MRSPS is likely multifactorial, with the patient’s CVADs playing a critical role. The dissections, particularly in the vertebral arteries, might be a cause of ischemia (7), leading to the hypoperfusion in the posterior circulation and bilateral temporal lobes (Figure 1B). This ischemia was related to the complex pain (8–10) and vertigo (11, 12), as seen in the patient following the successful therapy (Figure 1). The top-down progression of pain with vertigo might be attributed to the sequential involvement of the nervous system, starting from higher centers and progressively affecting lower regions, further supporting the mechanism of central pain (13).

### Mechanisms

Ischemic CVADs can lead to the prolonged and chronic cerebral hypoperfusion (Figure 1B), potentially inducing pain and/or vertigo through the following mechanisms such as the demyelination (14), white matter degeneration (15), sustained abnormal activity of sensory neurons (16), lipid mediators represented by lysophosphatidic acid (17), inflammatory responses (18), neurotransmitter imbalances (19), neuronal dysfunction (20), cerebral autoregulatory disorders (21), and crosstalk between the immune system and nociceptive neurons (22), peripheral and central sensitization (23–25), and so on. When different neurons and nerve roots are affected, corresponding symptoms, such as pain and/or vertigo, occur. The physical triggers, such as touch and movement, in exacerbating the pain suggests a possible neurosensory component (26). This might be associated with the sensitization of both peripheral and central nociceptive pathways (27), a phenomenon commonly observed in chronic pain conditions. To a large extent, the success of peripheral nerve damage treatment is contingent upon brain plasticity during recovery (28), which was well exemplified in our study. With the recovery of hypoperfusion, the patient’s symptoms of pain and dizziness have completely disappeared (Figures 1B,H).

### Treatment

The treatment strategy was guided by the principle of addressing the most significant pathology first. Given the relative severity and length of the right CVAD, it was identified as the primary target for therapy. The decision to take dual antiplatelets followed by stent implantation was based on the evidence supporting this approach in managing CVAD and improving perfusion (7, 29, 30). Following intervention, the diameter of the right vertebral artery has normalized (Figure 1A vs. Figure 1G), leading to an improvement in perfusion (Figure 1B vs. Figure 1H), and consequently, the patient’s symptoms have entirely resolved (Table 1). Therefore, stent reconstruction is especially important, as it targets not only the restoration of the dissecting artery integrity but also the potential mitigation of the ischemic brain insult (Figures 1F–H). This therapy aligns with the current viewpoint of the pathophysiology of MRSPS, where the improved perfusion is expected to alleviate its symptoms (Figure 1B vs. Figure 1H).

### Outcomes

The complete resolution of the patient’s MRSPS post-therapy was a favorable outcome. In particular, it suggests that the identified CVADs were indeed the primary drivers of MRSPS. This study provides valuable insights into the therapy of complex pain syndromes with vascular etiologies. It underscores the importance of a comprehensive diagnostic approach, including advanced imaging techniques like DCE-CT, which can reveal cryptogenic but clinically significant vascular abnormalities (Figures 1C–E). Especially noteworthy is the fact that the persistent stomach pain, which had been resistant to treatment, has been resolved after the stent therapy (Figure 1). Moreover, foods that used to provoke the pain can now be eaten without issue. Despite the successful therapy in this case (Table 1), further study is required to understand the full spectrum of MRSPS, including identifying other etiologies, exploring the genetic and environmental factors, and developing standardized diagnostic and therapeutic criteria.

### Conclusion

Hypoperfusion of brain tissue is one of the significant causes of MRSPS, and CVADs are a crucial pathological basis for this hypoperfusion. Repairing CVADs is an effective method for eliminating MRSPS.

## Data Availability

The original contributions presented in the study are included in the article/supplementary material, further inquiries can be directed to the corresponding authors.
